# Modeling and Performance Analysis of Opportunistic Link Selection for UAV Communication

**DOI:** 10.3390/s21020534

**Published:** 2021-01-13

**Authors:** Zhengjia Xu, Ivan Petrunin, Antonios Tsourdos

**Affiliations:** School of Aerospace, Transport and Manufacturing, Cranfield University, Cranfield MK43, UK; i.petrunin@cranfield.ac.uk (I.P.); a.tsourdos@cranfield.ac.uk (A.T.)

**Keywords:** cognitive communication, opportunistic spectrum access, UAV communication, air-to-ground communication, spectrum sharing, spectrum management, spectrum scarce operation

## Abstract

In anticipation of wide implementation of 5G technologies, the scarcity of spectrum resources for the unmanned aerial vehicles (UAVs) communication remains one of the major challenges in arranging safe drone operations. Dynamic spectrum management among multiple UAVs as a tool that is able to address this issue, requires integrated solutions with considerations of heterogeneous link types and support of the multi-UAV operations. This paper proposes a synthesized resource allocation and opportunistic link selection (RA-OLS) scheme for the air-to-ground (A2G) UAV communication with dynamic link selections. The link opportunities using link hopping sequences (LHSs) are allocated in the GCSs for alleviating the internal collisions within the UAV network, offloading the on-board computations in the spectrum processing function, and avoiding the contention in the air. In this context, exclusive technical solutions are proposed to form the prototype system. A sub-optimal allocation method based on the greedy algorithm is presented for addressing the resource allocation problem. A mathematical model of the RA-OLS throughput with above propositions is formulated for the spectrum dense and scarce environments. An interference factor is introduced to measure the protection effects on the primary users. The proposed throughput model approximates the simulated communication under requirements of small errors in the spectrum dense environment and the spectrum scarce environment, where the sensitivity analysis is implemented. The proposed RA-OLS outperforms the static communication scheme in terms of the utilization rate by over 50% in case when multiple links are available. It also enables the collaborative communication when the spectral resources are in scarcity. The impacts from diverse parameters on the RA-OLS communication performance are analyzed.

## 1. Introduction

Unmanned aerial vehicles (UAVs) are gaining popularity in civil, commercial, and military services due to their mobility, flexibility, and ease of deployment. Given the fact that without holding valid spectrum licenses, most UAVs operate on the industry, scientific, and medical (ISM) band [1] or a mixture of those bands. Lacking the monitoring of the spectrum utilization leading to more spectrum conflicts toward existing users, the supervision of UAV communication behavior is major challenge in the federal aviation administration (FAA) [2]. Meanwhile, the multi-link operational concept (MLOC) [3] applied for the heterogeneous aeronautical network, such as the dual links with SatComm and cellular link [4], and mmWave with cmWave [5], is promising to increase the communication resilience. Moreover, MLOC ensures the reliability of UAV operations at the cost of the occupying multiple heterogeneous link resources dynamically.

The conventional aeronautical communication including the backhaul transmission typically applies static link solutions. The aeronautical telecommunication network (ATN) standard regulated by the international civil aviation organization (ICAO)is mostly adopted to support the aircraft ground-to-ground (G2G), air-to-ground (A2G) and avionic data exchange [6]. The TCP/IP suite of protocols and open systems interconnection (OSI) protocol stack are used to form the prototype ATN. Some ATNs are highlighted, such as the controller pilot data link communications (CPDLC), automatic dependent surveillance (ADS), flight information service (FIS), and data link in aircraft communications addressing and reporting system (ACARS) [7]. Nevertheless, the above solutions are originally designed for licensed operators and the access procedures are complex for UAVs. Additionally, the typical ATN is difficult to embrace heterogeneous UAV platforms and multiple data link types resulted by the mission-driven feature. Some common links are highlighted: short-range links of Bluetooth, Wifi, Zigbee/Xbee, WiMAX, UWB; medium-range link of AeroMACs; and long or worldwide range services of GSM, LTE, SatComm. Given the fact that no dominant regulations exist in the diverse communication links [2], there is a need of re-thinking the current ATN solution and proposing an integrated communication solution for UAV control and non-payload communications (CNPC) data links.

To achieve the sufficient exploitation of spectrum resources and manage the collaborative communication among multiple UAVs, two feasible technologies in the cognitive communication are highlighted. The the opportunistic spectrum access (OSA) aims at addressing the spectrum access problem, while the spectrum sharing is for coordinating spectrum resources among multiple users. The above approaches are successfully applied in cognitive radio networks (CRNs), vehicle-to-everything (V2X), Internet of things (IoTs), and cellular networks [8].

OSA allows secondary users, i.e., UAVs in this case, to occupy spectrum resources in an opportunistic manner under a presumption of causing non-interference to primary users. As an interweave mode in CRN concept [9], typical OSA employs a listening-before-transmission mechanism [10], where the spectrum sensing phase is required before each transmission to protect PUs [10,11,12]. The authors in [11] proposed an efficient spectrum sensing enabled OSA scheme for the ultra-dense operations, and discussed the determination of spectrum processing time where the priority control mechanism is employed. The authors in [12] investigated the trade-off problem between the saturation throughput and sensing time, where the optimization of spectrum sensing time is addressed by fusing collaborative sensor outcomes.

Dynamic spectrum allocation or assignment schemes for the spectrum sharing is extensively studied especially under the TV bands [13]. The spectrum allocation can be grouped into two categories, i.e., the central authority based structure and distributed structure [14]. The centralized communication networks require a coordinator and are enabled with an exclusive common control channel for delivering the access or the scheduling model. For instance, authors in [13] presented a channel assignment scheme in the link layer utilizing the layer graph to achieve a global optimized performance. Authors in [15] presented a modified game theory approach for maximizing the spectrum resources on account of the priority of sensing data and reduction of spectrum handoff. On the other hand, the distributed spectrum allocation requires a reporting and negotiation phase before each transmission to tackle the contention problem and avoid internal collisions. Some methods are highlighted, such as the exhaustive search enabled overlapping algorithm for the contention resolution [16], joint pricing enabled with the game theory for the collaborative spectrum allocation [17], and a size-negotiable auction-based allocation scheme [18]. Given the constraint computation power in the UAV processors, the computation loads can be mitigated with the centralized communication scheme. In specific, the link scheduling and link collision avoidance are offloaded to the terrestrial stations from the airborne.

The media access control (MAC) controls the hardware and makes the frequency or channel hopping (link hopping in this case) possible. Several capabilities can be achieved in the MAC layer, such as the optimization of the energy consumption, overhearing, and channel idle listening mechanism for determining the presence of signals and the quality of service (QoS) improvement. Authors in [19] firstly presented a synchronous duty cycle management scheme of Sensor MAC for the energy optimization purpose. The authors in [10] proposed a two-layer MAC scheme, i.e., CR-ALOHA based MAC protocol for the OSA scheme by applying the random access scheme in selecting channels and considering imperfect spectrum detectors. Authors in [20] presented a MAC design for the mesh networks with an integration of the energy awareness and routine algorithm. However, the above works neither lack the consideration of the spectrum sharing scheme nor assume to implement the spectrum sharing on-board. Therefore, the offloaded spectrum sharing based MAC layer is needed.

As a cross-layer protocol initially designed for autonomous vehicles, MAVLink has been successfully used in several autonomous systems, e.g., APM, Pixhawk, DJI, Mikrokopter, YUNEEC, Micropilot, micro unmanned systems, and Parrot (Ar.unmanned system) [21]. Two mechanisms exist in the conventional MAVLink, i.e., the delivery of command, control and communication (C3) messages, and the heartbeat message for the periodic monitoring purpose. In our case, providing the offloaded spectrum sharing, the communication scheme generated in the ground control stations (GCSs) needs to be forwarded to UAVs, hence the MAVLink data structure also demands to be modified for our case.

With the above remarks, this paper aims at presenting a synthesized resource allocation and opportunistic link selection (RA-OLS) scheme for supporting multiple links and delivering command and control (C2) messages for the air-to-ground (A2G) communication. RA-OLS enables the dynamic management of RF resources and allows for the opportunistic access in a sequential manner so as to facilitate the UAV operation under the spectrum scarce environment. UAVs envision to have multiple transmitters along with spectrum detectors to identify the vacancy of the link to prevent the collision, while the link selection and scheduling is implemented in GCSs. Spatial and temporal spectral information is processed, and the link scheduling resolutions, i.e., link hopping sequences (LHSs), are generated by a link allocation algorithm. LHSs are packed with time labels into the MAVLink data structure, and uploaded to UAVs through a predefined common control forward link. Enabled with a link-hopping based MAC protocol, UAVs start the data transmission in accordance with the uploaded LHSs.

The RA-OLS is a synthesized solution which embraces multiple methods, e.g., the link selection, OSA, and resource allocation. Different from the traditional link selection research, the time-varying feature is considered, hence the link resources are modeled as link opportunities in the frequency and time domain. Distinct from the OSA enabled communication or the resource allocation enabled communication, this paper aims at addressing the problems of when which and how links with high quality of service (QoS) are used for the UAV operations. The contribution of this paper is to propose and model a centralized link sharing based communication solution for UAV applications, where the link allocation, link access, upload link effect, and spectrum sensing effect are considered. Several features are highlighted with the proposed RA-OLS scheme.

We derive a mathematical performance model in terms of throughput for the RA-OLS scheme which can be used for the spectrum dense and scarce environments. Several aspects are integrated in the model, such as the link allocation algorithm, decision fusion with multiple on-board spectrum detectors, re-sensing scheme considering non-ideal spectrum detectors, and packet dropout rate among common control links. A Markov based Gilbert-Elliott model is presented for estimating the packet drop in uplinks, where the Ricean fading is used for modeling the channel environment. Statistics theory is used in the derivation. Moreover, we present an interference factor (IF) as the outrage probability to measure how UAVs cause interference to other existing users.

A lightweight cross-layer protocol is proposed which is modified from the MAVLink for uploading the LHSs to UAVs. A spectrum sensing based MAC protocol is presented for enabling the opportunistic link selection with the re-sensing policy.

An efficient greedy based spectrum allocation algorithm is developed to accelerate the computation and enables the spectrum sharing with QoS and fairness fitting among UAVs.

The rest of this paper is organized as follows: Section 2 presented the proposed RA-OLS communication scheme along with the corresponding technical solutions. Section 3 denotes mathematical models towards the proposed RA-OLS system. Numeral simulations are presented in Section 4 following by the conclusions in Section 5.

## 2. System Architecture and Technical Design

**Figure 1:**
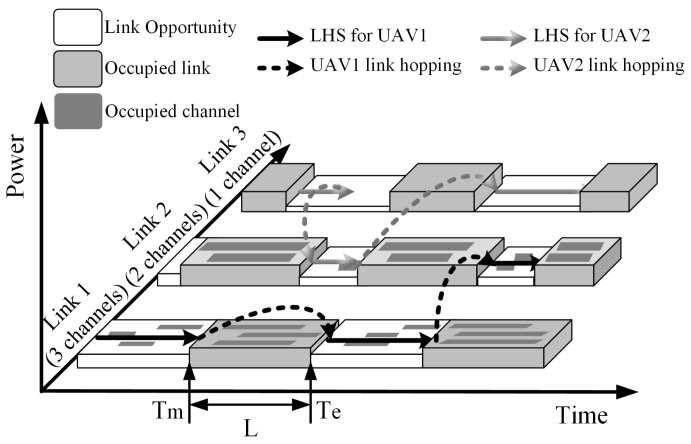
Demonstration of a spectrum occupation diagram enabled by the RA-OLS scheme.

A conceptual diagram for the RA-OLS scheme is shown in Figure 1. The PU traffic model which reveals the occupancy information in wide bandwidth is represented as a binary ON/OFF model [22]. Given the time-varying feature in the spectrum, white blocks form a link opportunity set *I* aiming to be used by UAVs. The dark blocks are the congested links that need to be avoided in the utilization. The solid lines with arrows are the desired LHS schemes T, and the virtual lines represent the link-hopping maneuver between *I*.

**Figure 2:**
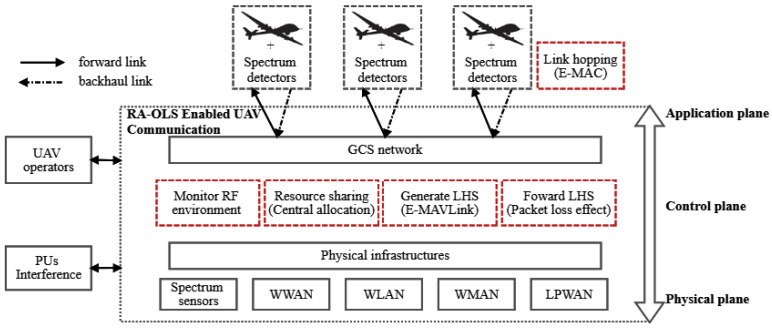
System diagram of the RA-OLS enabled UAV communication.

Enabled with the presented link-hopping concept, the RA-OLS based UAV communication diagram is illustrated in the Figure 2, where some fundamental components are: *K* UAVs equipped with *M* transmitters, GCS network, spectrum detectors deployed among UAVs for acknowledging the PU vacancy, spectrum sensors deployed in terrestrial for collecting the PU traffic pattern information, and some general terrestrial infrastructures denoting the existing heterogeneous networks, such as wireless wide area network (WWAN), wireless local area network (WLAN), wireless metropolitan area network (WMAN) and low power wide area network (LPWAN).

Given some unique features existing in the RA-OLS scheme, such as the offloaded spectrum processing, central resource management, and dynamic access to heterogeneous links, some exclusive functions are highlighted in red virtual boxes, for instance monitoring RF environment, resource sharing, creating LHSs, forwarding LHSs, and link hopping function in the airborne.

With an assumption that PUs are unknown in prior, the spectrum processing is critical to obtain the real-time PU traffic information in the function of monitoring RF environment, where several detection, identification, and prediction methods [23,24] were proposed. Based on the extracted PU features, the resource sharing function enables the collaborative occupation of spectrum resources among UAVs, where the contention issue is mitigated in such a centralized mechanism. Along with the C2 messages received from UAV operators, the LHSs are segmented into multiple frames, packed with the unified data structure, and forwarded to UAVs, where the packet dropout effect is critical because of unreliable upload links and lacking static backhaul link solutions. The link hopping function conducted among UAVs is to implement the data transmission through multiple links, whilst the link establishment and maintenance in the current link solutions are not considered.

In specific, three technical gaps are put forward: (1) how to transmit the link opportunity information to individual UAVs; (2) how to allocate link opportunities over multiple UAVs in an equal manner; (3) and what is the MAC layer like given the link hopping function. The technical solutions are provided to address the above gaps to form a prototype scheme, i.e., the extended MAVLink (E-MAVLink), the enhanced MAC (E-MAC), and the link resource allocation algorithm.

### 2.1. Extended MAVLink

**Figure 3:**
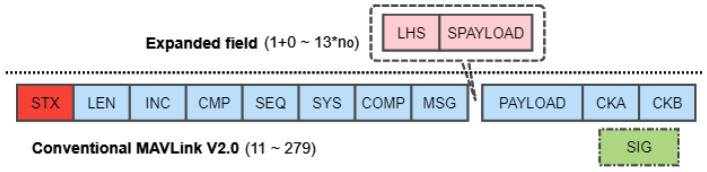
E-MAVLink Header.

We assume that the link access is triggered by the time and event, and link opportunities *I* are characterized by the start time $T_{m}$, end time $T_{e}$, duration of the link opportunity $L=T_{e}-T_{m}$, and a quality factor *Q*. Only $T_{m}$, $T_{e}$, and link identifier need to be uploaded to UAVs, while the link quality is estimated on-board with spectrum detectors. Multiple link opportunities are formed as an LHS. A completed LHS set may be separated into multiple data frames when the opportunity number exceeds the maximum number of $n_{o}$. Developed from the MAVLink protocol [21], the proposed E-MAVLink frame structure is presented in Figure 3 with its explanations clarified in the Table 1.

Distinct from the typical MAVLink protocol, two additional fields, i.e., NLHS and SPAYLOAD, are appended before the payload of data. The NLHS is the number of LHS packet in this frame, and SPAYLOAD represents the specific content of LHS messages consisting of a link identifier, start time stamp, and end time stamp. The link identifier has 1 byte size, hence the hardware limitation of $n_{o}$ is $n_{o}\in \left[0,255\right]$. The time stamps are unified into the same format with the fixed size of 6 bytes resulting in 13 bytes opportunity size. Consequently, the length of the E-MAVLink frame is extended from the traditional range of [11,279] [21] to $\left[12,279\right]+n_{o}\;\cdot \;13$ bytes.

### 2.2. Enhanced MAC

The proposed E-MAC is developed from a two-stage slotted CR-ALOHA MAC layer [10]. Some distinctions are highlighted, for instance, the slotted CR-ALOHA MAC demands a periodic detection of the PU presence, whilst such periodicity is not reflected in the RA-OLS scheme; the CR-ALOHA MAC develops the random access within the determined time intervals, whilst the link selection function is offloaded in GCSs; the data frame length is fixed in the CR-ALOHA, whilst it is dynamic according to the PU traffic pattern among links; the CR-ALOHA MAC implements the hopping between the frequency or channels in interest, while the E-MAC switches between diverse links enabled with multiple detectors and fusion method. Moreover, the re-sensing mechanism with the MAX-N-RS policy [25] is considered in the E-MAC with the consideration of non-ideal spectrum detectors.

**Figure 4:**
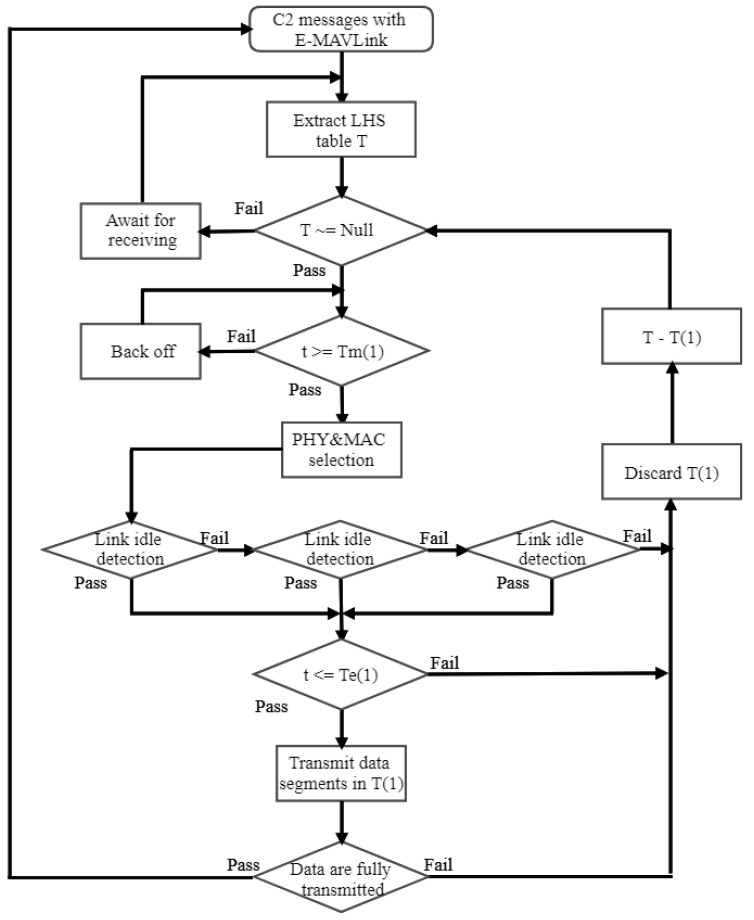
Enhanced MAC protocol enabled with the sensing before transmission scheme where the maximum re-sensing time R=3.

We define the symbol T as the extracted sets of link opportunities from LHSs with the labeled start time $T_{m}$ and end time $T_{e}$ as elements. T(1) represents the first element of T. *t* is the current processing time. The framework diagram of the proposed E-MAC layer is shown in Figure 4.

As depicted in Figure 4, the execution of E-MAC is processed as follows:

Considering the case of delay in retrieving the LHS set T, some LHSs are removed firstly following the equation: $T-f_{i}\left(T_{e}<=t\right)$, where the $f_{i}$ function is to find elements in the dataset, and the operation of ‘−’ denotes the removal of elements from the dataset.

The operation remains awaiting until it is triggered by the start time, where the time synchronization problem can be solved by the coordination function in SUs [10].

Given the collaborative sensing result with multiple spectrum detectors, UAVs detect the occupation status among links in interest before performing the transmission. If the link is determined to be idle, the request data will be transmitted within the remaining time. Otherwise, the spectrum detectors would keep sensing for the maximum of *R* times to acquire the availability of PUs.

Only when the current time *t* is within the opportunity duration, i.e., $t<=T_{e}\left(1\right)$, this opportunity is used, where *t* is affected by the re-sensing time $T_{sen}$ and the MAC processing time $\delta_{T}$.

It is noted that this E-MAC layer aims at addressing the problem of the time-event triggered link access with a unified scheme. Some unique link maintenance and establishment mechanisms, e.g., request to send (RTS), clear to send (CTS), and acknowledgment (ACK) [25] are negligible in the E-MAC design and analysis.

### 2.3. Link Resource Allocation

The allocation of link resources runs in GCSs and is critical in managing spectrum resources among multiple UAVs. Two objectives are considered in the allocation, i.e., the allocation of link opportunities with the best quality and maximizing the link occupation time among UAVs. As discussed in Section 2.1, the link opportunities are characterized by the start time $T_{m}$, end time $T_{e}$, and a quality factor *Q*, where *Q* denotes channel properties, e.g., scattering, fading, and power decay, and can be obtained from channel state information (CSI) through heartbeat messages. The duration of link opportunity is obtained: $L=T_{e}-T_{m}$. We define the utilization rate $U=\frac{\sum L}{T}$. The maximization of LHS T can be addressed by solving the following objective function:

(1) $$\begin{array}{cc} \; & \max_{T}S=\sum_{k=1}^{K}Q\left(T\right)U\left(k,T\right)\\ & e.g.,\;T\in \mathrm{dom}\{I\},k\in \mathrm{dom}\{K\} \end{array}$$

It is noting that when $T=T_{o}$ which means the time length for the optimization is equivalent to the time of predicting PU patterns, the communication reaches the maximal throughput values by using the spectral resources sufficiently.

Given the time-varying feature in the T, we firstly make the following assumptions before addressing the function (1): (1) each link opportunity can only be allocated to one UAV; (2) UAV cannot switch to another link before finishing the current opportunity; and (3) the link opportunity can be accessed intermediately.

This paper presents an achievable sub-optimal algorithm, i.e., the ϵ-greedy resource allocation algorithm developed from our previous work in [26]. Some advantages are highlighted, such as the low complexity, feasibility in adding constraints, and convenience in modeling.

The optimization of *U* is substituted with two cases: (1) minimizing the time interval ΔT between the start time $T_{m}$ and the end time $T_{e}$ of the resource in the s−1 step, and (2) maximizing the remaining time duration ΔL between the end time $T_{e}$ of the resource in the s−1 step and the end time $T_{e}$ of the resource in interest. Moreover, regarding the purpose of leveraging resources among multiple *K* UAVs, a fairness fitting factor is integrated by summarizing the allocated resource length $T_{b}=\sum_{s=1}^{s-1}\Delta L\left(k,s\right)$. To this end, a monotonous differential objective function at the iteration *s* for the $k_{th}$ UAV is formulated as:

(2) $$\begin{array}{cc} \; & \max_{T}\Delta S\left(k,s\right)=\lambda_{L}\Delta L\left(i\right)\lambda_{Q}Q\left(n\right)\lambda_{\Delta T}^{\Delta T(k,i,s)}\lambda_{b}^{\sum \left(T\left(k\right)\lambda_{Q}Q\right)}\\ & \;\;\;\;\;\;\Delta T=T_{m}\left(i\right)-T_{e}\left(T\left(k,s-1\right)\right)\\ & \;\;\;\;\;\;\Delta L=T_{e}\left(T\left(k,s\right)\right)-T_{e}\left(T\left(k,s-1\right)\right)\\ & \;\;\;\;\;\;\;T_{b}=\sum_{s=1}^{s-1}\Delta L(k,s)\\ & e.g.,\;T\in \mathrm{dom}\{I\},i\in \mathrm{dom}\{I\},n\in \mathrm{dom}\{N\},k\in \mathrm{dom}\{K\} \end{array}$$

where $\lambda_{\Delta T}$ and $\lambda_{b}$ represent the discount weight for minimizing the time gap and balancing allocation results among UAVs with values ranging in [0,1]. $\lambda_{L}$ and $\lambda_{Q}$ are the coefficient weights. *n* and *i* are the index of link {N} and set of link opportunities {I}, respectively. The $\sum \left(T\left(k\right)\lambda_{Q}Q\right)$ summarizes the resources weighted by $\lambda_{Q}$ for $k_{th}$ UAV.

Given a pre-known I(k) for the $k_{th}$ UAV, a general framework of the ϵ-greedy based link resource allocation strategy for one iterative processing is presented in the Algorithm 1 [26].

When having a small ϵ value, it might be possible that one link opportunity is allocated to multiple UAVs, thus the algorithm demands an avoidance mechanism to manage the spectrum and prevent collisions among UAVs. Specifically, only the UAV with the highest ΔS value is allocated to the opportunity. It is noted that the time effects, e.g., delay in the E-MAC progressing, and multi-sensing period, are not considered during the allocation phase. Moreover, given that one LHS consists of multiple iterative steps and the fairness fitting factor in the Equation (2) consists of an accumulative value of $\sum T\left(k,s-1\right)$, an appropriate selection of the accumulative length is critical to leveraging different effects in ΔS by way of periodic clearance of the template accumulative parameter.
**Algorithm 1** Pseudo-code of Greedy Based Algorithm for Time Sequence Resource Allocation1:Initialization of parameters2:**while**I(k)∉∅**do**3:     Calculate the differential objective function ΔS towards *k* UAV with the closest link opportunity in the time domain.4:     Select a flexible threshold value $T_{h}=\epsilon \;\cdot \;\max\left(\Delta S(l,s)\right)$ according to the maximum value of objective functions.5:     Find link opportunities for each UAV that satisfies $\Delta S>=T_{h}$.6:     **if** Only one UAV is allocated to this opportunity **then**7:         Save link opportunity T to $k_{th}$ UAV.8:         Remove elements in I(k) when $T_{e}\left(I\left(k\right)\right)<=T\left(s\right)$.9:         Update parameters.10:     **else**11:         Save link opportunities T with the maximum ΔS value to $k_{th}$ UAV.12:         Remove elements in I(k) when $T_{e}\left(I\left(k\right)\right)<=T\left(s\right)$.13:         Update parameters.14:     **end if**15:**end while**16:**return**T

## 3. Modeling and Analysis

Due to lacking theoretical models for allocation algorithms, especially with sub-optimal algorithm, this section aims at presenting a mathematical model for RA-OLS with unknown spectrum allocation performance. Moreover, several aspects are considered in the model, e.g., the imperfect spectrum detectors with the probability of false alarm and miss detection, the unreliable forward links resulting in packet dropoutes, and delays in the E-MAC leading to the repeat sensing of PU states. A saturation throughput is modeled, along with an interference factor (IF) presented to measure the interference degree towards other PUs.

We assume that the active (run) state A in the PU transmission and the inactive state (burst) state I both follow exponential distributions [27] with the cumulative distribution functions (CDFs) of $Pr_{A}\left(N\left(t\right)=0\right)=1-e^{-\lambda_{a}t}$ and $Pr_{I}\left(N\left(t\right)=0\right)=1-e^{-\lambda_{v}t}$, where $\lambda_{a}$ and $\lambda_{v}$ are the averaged active length and inactive length of the PU traffic, respectively. The estimation for $\lambda_{a}$ and $\lambda_{v}$ can be done by observing the occurrence of events through estimation methods, such as the moment estimation, maximum likelihood estimation (MLE), and least square estimation (LSE) [28]. We assume that the arrival of link opportunities in the LHS set T with the link allocation algorithm also follows a Poisson distribution. The probability mass function (PMF) of the link opportunity occurrence is denoted by $Pr_{T}\left(C_{1}\left(t\right)=n\right)=\frac{{\left(\lambda_{F}t\right)}^{n}e^{-\lambda_{F}t}}{n!}$, where $\lambda_{F}$ is regarded as the average number of spectrum opportunity. Owing to the fairness fitting function, UAVs share the same Poisson distribution with $\lambda_{F}$, Therefore, we can estimate the number of spectrum opportunities *F* in one LHS schemes as: $F=E[Pr_{T}\left(C_{1}\left(t<T_{o}\right)\right]=\lambda_{F}T_{o}$.

Moreover, we assume that the actions of UAV access to links are independent and follow the Poisson process, where the occurrence of the link opportunities *I* for all the UAVs satisfies the same $\lambda_{F}$ Poisson distributions owing to the fairness fitting function. Other time-delay effects, such as carrier sensing time, and link hopping time, are modeled as a fixed processing time $\delta_{T}$.

### 3.1. Throughput Model

Due to the additional integration of spectrum sensing before transmission mechanism, a successful transmission of data packets considers two aspects: (1) LHS is successfully received by UAVs through the forward links; (2) spectrum detectors equipped on UAVs correctly identify the spectrum occupancy of PU signals.

#### 3.1.1. Packet Dropout

To enable a reasonable estimation of packet dropout probability for UAV communications, Gilbert-Elliott (GE) [29] is typically applied with a two-state discrete Markov model in modeling wireless A2G aeronautical data links [30]. Compared with the Bernoulli process model [31], GE considers the correlation of packet dropout patterns which are caused by failure in receivers and channel conditions (multi-path effect is dominant in UAV applications).

**Figure 5:**
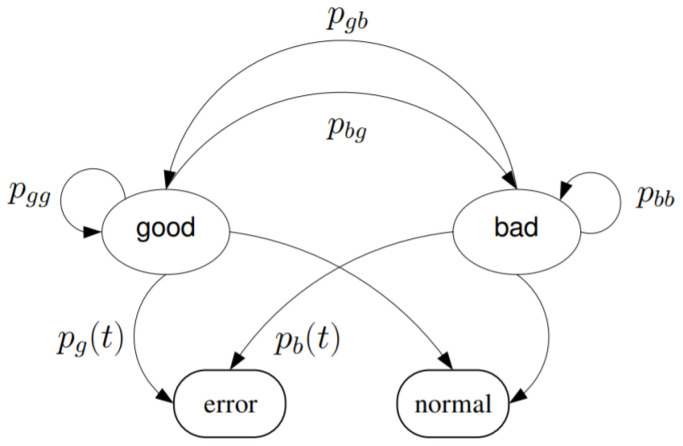
State transition diagram based on a two-state Markov process [32].

In this work, we use the Markov based GE model presented in [32] owing to its incorporation of Ricean fading effect and the time-varying nature of the packet dropout. Given two states are modeled, i.e., good (labeled by a subscript of g) and bad (labeled by a subscript of b), the Markov process presented in Figure 5 is characterized by probabilities, e.g., $p_{gg}$, $p_{gb}$, $p_{bb}$ and $p_{bg}$. The $p_{xy}$ format denotes the probability of the state switching from x to y. Let $\pi_{g}$ and $\pi_{b}$ denote the stationary distributions for the good state and bad state, respectively. The following equations are obtained:

(3) $$p_{gg}=1-p_{gb},p_{bb}=1-p_{bg};\pi_{g}=\frac{p_{bg}}{p_{gb}+p_{bg}},\pi_{b}=\frac{p_{gb}}{p_{gb}+p_{bg}}$$

Given the Ricean fading model can be represented by the Rice distribution, the received signal amplitude is characterized with the following two parameters:

(4) $$v^{2}=\frac{\kappa \Omega }{1+\kappa },\omega^{2}=\frac{\Omega }{2(1+\kappa )}$$

where κ denotes the proportion of the direct path effect to the scattered path effect. Ω denotes the received signal power, which is impacted by the distance *d* and the path loss via the direct link:

(5) $$\Omega =\Omega_{0}{\left(\frac{d}{d_{0}}\right)}^{-\eta }$$

where $\Omega_{0}$ denotes the signal power received at the reference distance of $d_{0}$. η is the path loss coefficient and is reasonably configured to 2 in UAV applications (corresponding to the free space model).

With a determined receiver sensitivity $S_{r}$, the probability for the time sensitive error is denoted:

(6) $$p_{g}\left(t\right)=1-Q\left(\frac{v}{\omega },\frac{\sqrt{2S_{r}}}{\omega }\right)$$

where *Q* is the Marcum Q-function.

The estimated packet dropout rate $P_{d}$ hence is denoted:

(7) $$P_{d}=\varphi_{0}\pi_{g}+\pi_{b}$$

where $\varphi_{0}=\frac{1}{N_{p}}\sum_{t}p_{g}\left(t\right)$ and $N_{p}$ is the number of observations.

Consequently, the expectation of the opportunity number per each upload is denoted by:

(8) $$E\left[N_{d}\right]=\left(f_{c}\left(n_{e}\right)-1\right)\left(1-P_{d}\right)n_{o}+\left(1-P_{d}\right)f_{c}\left(f_{rem}\left(\lambda_{F}T_{o},no\right)\right)$$

where $n_{e}$ represents the number of E-MAVLink frames for one LHS which is estimated by $n_{e}=F/n_{o}=\lambda_{F}T_{o}/n_{o}$; $f_{c}$ function rounds the element to the nearest value which is greater than itself; and $f_{rem}$ obtain the remainder after division.

#### 3.1.2. Spectrum Sensing

Due to having the spectrum sensing mechanism before transmission and heterogeneous link types, this section investigates a unified spectrum detection method, i.e., energy detection [33,34] for identifying the availability of PUs. The main concept of the energy detection is to summarize the energy of samples over the bandwidth in interest and compare with a threshold.

Given multiple sub-channels may exist, such as IEEE 802.11 [35], we investigate the fusion of multiple narrow-band spectrum detectors defined as $D\left(m,n\right)$, where *m* is the index of links with the overall *M* link number and *n* is the index of *N* channels. Hence, the detection of the PU signals among narrow-band channels over heterogeneous links can be formulated as $s\left(m,n,t\right)$. A binary hypothesis testing model for denoting ON/OFF status of the PU signals is hence developed from [33]:

(9) $$y\left(m,n,t\right)=\left\{\begin{array}{cc} n_{i}\left(m,n,t\right) & :H_{0}\\ h\left(m,n,t\right)s\left(m,n,t\right)+n_{i}\left(m,n,t\right) & :H_{1} \end{array}\right.$$

where $y\left(m,n,t\right)$ is the observed samples over the $m_{th}$ link and is received by the detector $D\left(m,n\right)$. $s\left(m,n,t\right)$ is the sample of PU signals. $h\left(m,n,t\right)$ is the channel gain between each detector and PUs [36]. $n_{i}\left(m,n,t\right)$ is the noise according to AWGN model. *t* is the selected sensing time. $H_{0}$ is the event that no signal exists, and $H_{1}$ represents the existence of PU signals.

Under a zero-mean circular symmetric complex Gaussian (CSCG) random vector and AWGN noise model [37], the detection probability $P_{D}$ over the single narrow-bandwidth can be developed from [33]:

(10) $$\begin{array}{cc} P_{D}\left(m,n\right) & =Pr\left(y\left(m,n,t\right)>\lambda_{D}\left(m,n\right);H_{1}\right)\\ & =Q\left(\frac{\lambda_{D}\left(m,n\right)-N\left(\sigma_{s}{\left(m,n\right)}^{2}+\sigma_{n}{\left(m,n\right)}^{2}\right)}{\sqrt{2N{\left(\sigma_{s}{\left(m,n\right)}^{2}+\sigma_{n}{\left(m,n\right)}^{2}\right)}^{2}}}\right). \end{array}$$

where $\sigma_{s}$ stands for the signal variance among single channels, and $\lambda_{D}$ denotes the threshold for determining the ON or OFF state.

The probability of false alarm $P_{F}$ is denoted as:

(11) $$\begin{array}{cc} P_{F}\left(m,n\right) & =Pr\left(y\left(m,n,t\right)>\lambda_{D}\left(m,n\right);H_{0}\right)\\ & =Q\left(\sqrt{N}\frac{\lambda_{D}\left(m,n\right)-\sigma_{n}{\left(m,n\right)}^{2}}{\sigma_{n}{\left(m,n\right)}^{2}}\right). \end{array}$$

Given the more concerns on the probability of false alarm than the detection probability, the decision policy employs a constant detection rate (CDR) criterion in this paper. A central chi-square distribution *Q* [33] assumption is applied, and *y* assumes to be approximately Gaussian distributed. For a given detection probability ${\bar{P}}_{D}$, the threshold value $\lambda_{D}$ for the narrow channel *n* over link *m* can be defined [33] as:

(12) $$\lambda_{D}\left(m,n\right)=\sigma_{n}{\left(m,n\right)}^{2}Q^{-1}\left({\bar{P}}_{D}\left(m,n\right)\right)\sqrt{\frac{2\sigma_{s}{\left(m,n\right)}^{2}+\sigma_{n}{\left(m,n\right)}^{2}}{N\sigma_{n}{\left(m,n\right)}^{2}}}+\sigma_{s}{\left(m,n\right)}^{2}+\sigma_{n}{\left(m,n\right)}^{2}$$

where $\sigma_{n}$ is the standard deviation of noise, *Q* is the Gaussian Q-function following the distribution of $Q\left(x\right)=\frac{1}{\sqrt{2\pi }}\int_{x}^{\infty }e^{-s^{2}/2}ds$, and $N=F_{s}\;\cdot \;T_{sen}$ represents the sample length induced for the estimation within the $T_{sen}$ duration.

Given the allowance of multiple *n* detectors to make decisions of the link occupancy over *m* links and fuse $N_{n}$ decisions into one, a k-out-of-N decision fusion rule [33] which is one typical hard fusion policy in the collaborative sensing is considered by agreeing with the decision when individual detector number reaches $N_{k}$. Consequently, the fused detection probability ${\hat{P}}_{D}$ and the probability of false alarm ${\hat{P}}_{F}$ with multiple detectors working on the link *m* thus are expressed as [33]:

(13) $${\hat{P}}_{un}\left(m\right)=\sum_{i=N_{k}}^{N_{n}}C\left(i,N_{n}\right)\prod_{j=1}^{i}P_{un}\left(m,j\right)\prod_{j=i+1}^{N_{n}}\left(1-P_{un}\left(m,j\right)\right)$$

where $un{=}^{\prime }D^{\prime }$ denotes the detection probability, and $un{=}^{\prime }F^{\prime }$ denotes the probability of false alarm.

It is worth noting that with the CDR criteria, the detection probability ${\hat{P}}_{D}\left(m\right)$ is determined according to the design requirements. Therefore, the probability of false alarm ${\hat{P}}_{F}\left(m\right)$ is one major concern affecting the detector performance.

#### 3.1.3. Saturation Throughput

We refer to the saturation throughput [38] which is a typical way to measure end-to-end MAC performance. In the saturation throughput analysis, we have a reasonable assumption that users continuously send C2 messages per every $T_{o}$ second.

We consider three effects when denoting the saturation throughput model, i.e., the uplink packet loss discussed in Section 3.1.1, opportunity loss considering the time delay in non-ideal spectrum detector, and miss detection in the sensing phase.

Let $C_{3}$ denote the case that UAV detects one link opportunity by mistake. We assume that the re-sensing mechanism follows a binomial distribution. Suppose the Max-N-RS scheme is employed in the E-MAC design discussed in Section 2.2, the PMF of the $C_{3}$ case over the $T_{i}\left(k\right)$ link opportunity within the re-sensing times *R* is denoted as:

(14) $${\hat{Pr}}_{a}\left(T_{i}\left(C_{3}=r,k\right)\right)=\left(\begin{array}{c} R\\ r \end{array}\right){\hat{P}}_{F}{\left(T_{i}\left(k\right)\right)}^{r}{\left(1-{\hat{P}}_{F}\left(T_{i}\left(k\right)\right)\right)}^{R-r}$$

where *r* is the index of access behaviours.

Given the maximum of *R* times for the re-sensing, the probability of missing detection of link opportunity for the $k_{th}$ UAV denotes ${\hat{Pr}}_{a}\left(C_{3}=R,k\right)={\hat{P}}_{F}{\left(T_{i}\left(k\right)\right)}^{R}$.

To simply the derivation of throughput, we assume that the links and detectors are homogeneous over the transmission rate and detection probability. Consider the time delay in the sensing phase may result in a smaller number of re-sensing, the possibility that the selected spectrum opportunity has been removed by the E-MAC denotes:

(15) $$Pr_{I}\left[N\left(t>=T_{D}\right)\right]=ReLu\left(\frac{\lambda_{F}T_{o}K-\left(n_{t}-e^{T_{D}\lambda_{v}}\right)}{\lambda_{F}T_{o}K}\right)$$

where $n_{t}=\frac{T_{o}M}{1/\lambda_{v}+1/\lambda_{a}}$. $T_{D}$ is the time delay which is modeled by $T_{D}=\delta_{T}+T_{sen}{\hat{P}}_{F}R$. The ReLu function denotes rectified linear unit (ReLu) and has the formulation of ReLu(x)=max(0,x).

We apply the order statistic theory [39] to estimate the possibility. With assumptions that opportunity length *L* is independent and identically distributed (i.i.d), we firstly re-order $n_{t}$ number of opportunities in the ascending order. Given the exponential distribution of random variables, the expectation of the first *n* variable is denoted by $E\left[X_{\left(n\right)}\right]=\frac{1}{\lambda }\left(1+\frac{1}{2}+\frac{1}{3}+\ldots +\frac{1}{n}\right)\approx \frac{\log n}{\beta }$. Therefore, the maximum opportunity number which has longer duration than $T_{D}$ denotes $n_{t}-exp\left(T_{D}\lambda_{v}\right)$. Given the total received number $\lambda_{F}T_{o}K$, a ReLu function is needed to ensure a positive possibility value.

We define E[L] as the averaged time duration for link opportunities without the time delay effect, as well as $E[L_{a}]$ for the link duration with time delays. Therefore, the expectation of $L_{a}$ after the E-MAC layer is denoted by:

(16) $$\begin{array}{cc} \; & \frac{E[L_{a}]}{E[L]}=\frac{\int_{T_{D}}^{+\infty }x\lambda_{v}e^{-\lambda_{v}x}dx}{\int_{0}^{+\infty }x\lambda_{v}e^{-\lambda_{v}x}dx}\\ & E\left[L_{a}\right]=E\left[L\right]\left(\lambda_{v}T_{D}+1\right) \end{array}$$

With the combination of (8), (13), (15), and (16), the saturation throughput model ${\hat{Th}}_{UAV}$ with considerations of E-MAC time delay, non-ideal on-board detection and packet dropout is given by:

(17) $$\begin{array}{cc} {\hat{Th}}_{UAV} & =\frac{\bar{G}E\left[N_{d}\right]\left(1-Pr_{I}\left(N\left(t>=T_{D}\right)\right)\right)\left(1-{\hat{Pr}}_{a}\left(C_{3}=R\right)\right)\left(E\left[L_{a}\right]-T_{D}\right)}{T_{o}}\\ & =\bar{G}\left(f_{c}\left(n_{e}\right)-1\right)\left(1-P_{d}\right)n_{o}+\left(1-P_{d}\right)f_{c}\left(f_{rem}\left(\lambda_{F}T_{o},no\right)\right)\left(1-ReLu\left(\frac{\lambda_{F}T_{o}K-\left(n_{t}-e^{T_{D}\lambda_{v}}\right)}{\lambda_{F}T_{o}K}\right)\right)\\ & \;\cdot \;\frac{\left(1-{\hat{P}}_{F}{\left(T_{i}\left(k\right)\right)}^{R}\right)E\left[L\right]\left(\lambda_{v}\left(\delta_{T}+T_{sen}{\hat{P}}_{F}R\right)+1\right)}{T_{o}} \end{array}$$

where $\bar{G}$ is the theoretical throughput with static connections.

With the acknowledge of $\lambda_{F}$ which can be calculated in statistic by the MLE method [28], another challenge is the estimation of E[L]. We consider two circumstances according to the uncertain relationship between the UAV number *K* and the link number *M*. When the UAV number *K* is bigger than the link number *M*, we define such as the spectrum scarce environment. All link opportunities aim to be allocated by the allocation algorithm. Therefore, the allocated opportunity length still follows the exponential distributions, hence $E\left[L\right]=\frac{1}{\lambda_{v}}$.

When the UAV number *K* is smaller than the link number *M*, which is defined as the spectrum dense environment, only the top *K* opportunities aim to be used during each allocation iterative. Similar with the proof in Equation (15), we use the statistics theory and the $o_{th}$ order statistics formulation is denoted by $E\left[L\right]\left(o\right)=\sum_{i=M-o+1}^{M}\frac{1}{i}$ [39]. Therefore, the average opportunity length is denoted as $E\left[L\right]=\frac{\sum_{j=M-K+1}^{M}\left(1/j\right)}{K\lambda_{v}}$. Consequently, the integrated throughput model per each UAV can be rewritten as a piece-wise function:

(18) $${\hat{Th}}_{UAV}=\left\{\begin{array}{cc} Th/\lambda_{v} & :M<=K\\ Th\;\cdot \;(\frac{\sum_{n=M-K+1}^{M}\sum_{j=M-n+1}^{M}\left(1/j\right)}{K\lambda_{v}}\left(\lambda_{v}T_{D}+1\right)-T_{D}) & :M>K \end{array}\right.$$

where $Th=\bar{G}\lambda_{F}{\left(1-P_{d}\right)}^{\lambda_{F}T_{o}/n_{o}}\left(e^{-\lambda_{v}T_{D}}-{\hat{P}}_{F}{\left(T_{i}\left(k\right)\right)}^{R}\right)$.

However, by simulations, we find that a leap between two piece-wise equations exists. Meanwhile, each iterative may not always allocate top *K* resources at each time especially when K=M. Therefore, we fine-tune this model with an empirical equation by introducing a more accurate factor between spectrum resources and the user number. The fine-tuned throughput model is denoted by:

(19) $${\hat{Th}}_{UAV}=\left\{\begin{array}{cc} Th/\lambda_{v} & :M<=K\\ Th\;\cdot \;(\frac{\sum_{n=M-K+1}^{M}\sum_{j=M-n+1}^{M}\left(1/j\right)}{K\lambda_{v}}\left(\lambda_{ca}^{\frac{\lambda_{v}M}{KC_{a}}}\left(\lambda_{v}T_{D}+1\right)+1\right)-T_{D}) & :M>K \end{array}\right.$$

where $C_{a}$ and $\lambda_{ca}$ are the coefficient weights for the adjustment purpose. $\lambda_{ca}<1$ which means that when spectrum resources are in relevantly scarcity ($\frac{\lambda_{v}M}{KC_{a}}$ is large), the throughput tends to move closer to the case of M<=K with a shorter opportunity duration. Reversely, under the spectrum dense environment, the opportunity duration increases.

Considering the fact that the re-sensing number in the E-MAC layer is always bigger than one. Therefore, the following function is needed when estimating the re-sensing effect on the E-MAC:

(20) $$T_{D}=\left\{\begin{array}{cc} \delta_{T}+T_{sen} & {\hat{P}}_{F}\left(T_{i}\left(k\right)\right)R<=1\\ \delta_{T}+T_{sen}{\hat{P}}_{F}\left(T_{i}\left(k\right)\right)R & 1<{\hat{P}}_{F}\left(T_{i}\left(k\right)\right)R<=R \end{array}\right.$$

Similarly, in the Equation (18), the transmission number $\lambda_{F}T_{o}/n_{o}$ in the forward link should be substituted with the following function to guarantee that at least one transmission via the forward link is performed:

(21) $$\lambda_{F}T_{o}/n_{o}=\left\{\begin{array}{cc} 1 & \lambda_{F}T_{o}/n_{o}<1\\ \lambda_{F}T_{o}/n_{o} & \lambda_{F}T_{o}/n_{o}>=1 \end{array}\right.$$

### 3.2. Interference Measurement

Regarding the fact that non-ideal spectrum detectors could generate interference to existing PU users, this section discusses one measurement model IF from [10] for revealing the interference extent. Two interference cases are considered, i.e., the missed detection of PU signal may generate collisions towards existing PU activities, and the burst occurrence of PU signal may be disturbed during the UAV transmission.

First, we assume that the unknown PU communication system employs a one-unit system [27], which means that one disruption in the transmission would lead to the failure of the whole system. We use hypothesis of $H_{2}$ and $H_{3}$ to denote the inactive and active states of PU models [10], and the PU occurrence model is denoted:

(22) $$Pr_{u}\left(m\right)=\left\{\begin{array}{cc} \lambda_{v}\left(m\right)e^{-\lambda_{v}\left(m\right)T_{sen}\left(m\right)}/\left(\lambda_{a}\left(m\right)+\lambda_{v}\left(m\right)\right) & :H_{2}\\ 1-\lambda_{v}\left(m\right)e^{-\lambda_{v}\left(m\right)T_{sen}\left(m\right)}/\left(\lambda_{a}\left(m\right)+\lambda_{v}\left(m\right)\right) & :H_{3} \end{array}\right.$$

where $T_{sen}$ denotes the length of sensing duration.

Providing the maximum *R* time of the re-sensing and *F* number of link opportunities in one LHS sagement, the measurement of the first case, i.e., the missed detection leading to the interference is formulated by combining (11) with (22): $IF_{1}=\left(1-{\bar{P}}_{D}{\left(m\right)}^{R\lambda_{F}}\right)Pr_{u}\left(m|H3\right)$.

For measuring the the second interference case, we define $Pr_{c}$ as the probability that PU awakes during the UAV transmission $E\left[L_{a}\right]-T_{D}$ after satisfying the $Pr_{u}\left(m|H2\right)$ assumption. Hence, the $Pr_{c}$ is denoted: $Pr_{c}\left(m\right)=\lambda_{v}\left(m\right)e^{-\lambda_{v}\left(m\right)T_{sen}\left(m\right)}/\left(\lambda_{a}\left(m\right)+\lambda_{v}\left(m\right)\right)-\lambda_{v}\left(m\right)e^{-\lambda_{v}\left(m\right)\left(E\left[L_{a}\right]-T_{D}\right)}/\left(\lambda_{a}\left(m\right)+\lambda_{v}\left(m\right)\right)$. The interference factor for the second case is thus given as: $IF_{2}=\left(1-{\hat{P}}_{F}{\left(m\right)}^{R\lambda_{F}}\right)Pr_{c}\left(m\right)$.

Consequently, the integrated interference factor IF which denotes the transmission collision degree over PUs is formulated:

(23) $$\begin{array}{cc} IF(m) & =IF_{1}+IF_{2}\\ & =\left(1-{\bar{P}}_{D}{\left(m\right)}^{R\lambda_{F}}\right)Pr_{u}\left(m|H3\right)+\left(1-{\hat{P}}_{F}{\left(m\right)}^{R\lambda_{F}}\right)Pr_{c}\left(m\right)\\ & =\left(1-{\bar{P}}_{D}{\left(m\right)}^{R\lambda_{F}}\right)\left(1-\lambda_{v}\left(m\right)\frac{e^{-\lambda_{v}\left(m\right)T_{sen}\left(m\right)}}{\lambda_{a}\left(m\right)+\lambda_{v}\left(m\right)}\right)+\\ & \frac{\lambda_{v}\left(m\right)}{\lambda_{a}\left(m\right)+\lambda_{v}\left(m\right)}\left(1-{\hat{P}}_{F}{\left(m\right)}^{R\lambda_{F}}\right)\left(e^{-\lambda_{v}\left(m\right)T_{sen}\left(m\right)}-e^{-\lambda_{v}\left(m\right)\left(E\left[L_{a}\right]-T_{D}\right)}\right) \end{array}$$

where $E[L_{a}]$ is discussed in the Section 3.1.3.

From the Equation (23), it is noting that with the increment of the $T_{sen}$ value, $IF_{1}$ and the component of $1-{\hat{P}}_{F}{\left(m\right)}^{R\lambda_{F}}$ in the $IF_{2}$ increase monotonically, and those factors relate to the performance of the airborne spectrum detector. However, the $Pr_{c}\left(m\right)$ in the $IF_{2}$ is non-monotonic because of the uncertain relation between $e^{-\lambda (m)}$ and $\frac{\partial E[L_{a}-T_{D}]}{\partial T_{sen}}$. Moreover, several parameters also present effects on the weights for separate components, which drive the needs for further analysis by simulations.

### 3.3. Analysis

We regard the typical static communication scheme as the benchmark, meaning that the dynamic hopping is not enabled, and SUs transmission is paused until PUs are in vacant. Therefore, the capacity of the static communication system is:

(24) $$C=\sum_{i=1}^{M}\left(C_{i}\right)$$

where $C_{i}$ is the maximum user number for $i_{th}$ link.

Facilitated by the coordination of individual link opportunities, the capacity for the RA-OLS communication can be maximized by allocating one opportunity to one UAV (no minimum throughput requirements):

(25) $$C=T_{o}\sum_{i=1}^{M}\left(\frac{T_{o}}{1/\lambda_{vi}+1/\lambda_{ai}}C_{i}\right)$$

where $\lambda_{vi}$ denotes to have $\lambda_{v}$ opportunity number for the $i_{th}$ link.

We also have the definition of the utilization rate $U=\frac{\sum L}{T_{o}}$ discussed in the Section 2.3, hence the best *U* averaged with *K* UAVs for the static scheme solution is estimated by:

(26) $$U\approx \frac{1/\lambda_{v}}{K(1/\lambda_{v}+1/\lambda_{a})}$$

where the negative effects, such as the packet dropout, non-ideal detection and delay in E-MAC, are not included.

For the RA-OLS scheme, the achievable utilization rate is obtained from the Equation (18):

(27) $$U={\hat{Th}}_{UAV}/\bar{G}$$

As presented in the throughput Equation (18), the throughput decreases monotonically with the increment of $T_{o}$ because the forward link model is not considered and more forward packages (longer E-MAVLink and more E-MAVLink frames) are delivered to UAVs leading to the bigger failure in the packet dropout.

Moreover, we group other parameters from the throughput Equation (18) into four categories, i.e., the PU pattern representatives denoted by *M*, *K*, $\lambda_{v}$ and $\lambda_{a}$, the non-ideal detector denoted by $\sigma_{s}$, $\sigma_{n}$, $N_{k}$, $F_{s}$ and $T_{sen}$, the time delay in E-MAC denoted by $\delta_{T}$, $T_{sen}$ and *R*, and the E-MAVLink length denoted by $n_{o}$. With the determined $\lambda_{F}$ values, the optimization of the throughput function for parameters can be done numerically. With the consideration of the IF function (23), the trade-off between throughput and interference effect might be challenging, especially with uncertain $\lambda_{F}$. The examination of the time delay effect on the E-MAC processing can be done by differing $T_{sen}$ only with the fixed $\delta_{T}$. Considering the difficulty in having $\lambda_{F}$ models, the throughput performance affected by parameters needs to be analyzed, as well as the IF result.

## 4. Experiments and Analysis

This section aims at evaluating the proposed RA-OLS communication scheme from the link perspective. Firstly, we demonstrate the communication with multiple heterogeneous links for single and multiple UAV operations. Afterwards, by using the Mento-Carlo method and developing a RA-OLS simulation, the performance for the mathematical model is analyzed in terms of the saturation throughput (in Section 3.1.3) along with the sensitivity analysis. The RA-OLS performance with IF is presented. The comparison analysis with the static communication is implemented.

### 4.1. Simulation for Practical Communication

According to the typical wireless communications discussed in [40], this paper considers three promising wireless communication links, i.e., Wi-Fi, Lora, and Sigfox operating on different frequency range. According to discussions in [40,41,42,43,44], we choose the reasonable configurations in Table 2. The reason of having multiple links is to embrace multiple UAV communication technologies for supporting the central management of UAV communication behaviors. Moreover, the dynamic communication via multiple links enables the frequency hopping to improve the communication reliability and flight safety when some frequency resources are congested, e.g., operation in urban areas.

**Figure 6:**
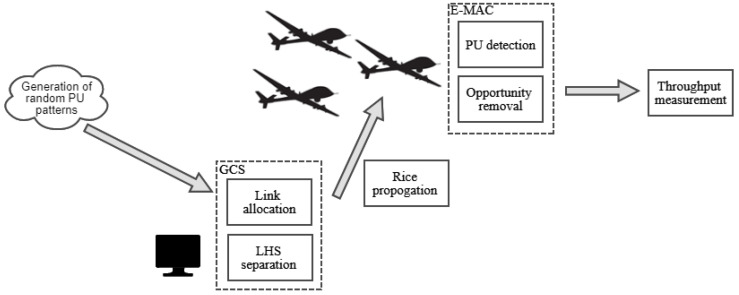
Simulation flow chart for dynamic spectrum sharing with RA-OLS scheme.

The developed RA-OLS simulator flow chart is presented in Figure 6. In the RA-OLS simulation, the RF environment, i.e., PU traffic patterns are randomly generated following the exponential distributions in the vacant and busy states. The propagation in the uplink, which could lead to the packet dropout adopts the Rice propagation model. When detecting the PU existence, AWGN noises are added to evaluate the on-board spectrum sensing performance. The link resources are allocated by the algorithm presented in Section 2.3. The detailed explanations for Figure 6 are presented in Algorithm 2.

The implementation of random timing sequences for a Poisson process in steps (3) and (4) refers to the Knuth algorithm [45]. The next time of having a random exponential distribution is $time=\frac{-\ln\left(rand\right)}{\lambda }$, where rand denotes a normalized random value, whose elements are uniformly distributed in [0,1].

It is noting that a successful UAV transmission applies only when three flags, i.e., $flag_{t}$ denoting the packet dropout, $flag_{s}$ denoting that the opportunity is detected, and $flag_{f}$ denoting that the opportunity duration is longer enough are all equivalent to 1. Therefore, the simulated saturation throughput per each UAV can be calculated in statistic following the equation:

(28) $$Th_{UAV}=mean_{k\in K}\left(\frac{\sum_{i\in T_{i}\left(k\right)}\left(flag_{t}\left(i\right)\;\cdot \;flag_{s}\left(i\right)\;\cdot \;flag_{f}\left(i\right)\;\cdot \;\bar{G}\left(i\right)\left(L\left(i\right)-T_{sensecount}\;\cdot \;T_{sen}-\delta_{T}\right)\right)}{T_{o}}\right)$$

where *i* is the index of the link opportunity $T_{i}$, and $T_{sensecount}$ is the simulated re-sensing time. The throughput is averaged among *K* number of UAVs with the mean function.

**Algorithm 2** Pseudo-code of RA-OLA Scheme for Random PU Traffic Patterns.
1:Initialization of parameters2:**while**m∈{M}**do** // Create PU traffic patterns.3:     Generate random ON model with $\lambda_{a}$ exponential distribution for $m_{th}$ link within $T_{o}$.4:     Generate random OFF model with $\lambda_{v}$ exponential distribution for $m_{th}$ link within $T_{o}$.5:
**end while**
6:Calculate probability of false alarm ${\hat{P}}_{F}\left(m\right)$ following Equation (13) given determined ${\hat{P}}_{D}$.7:Generate LHS T for each UAV following the allocation Algorithm 1.8:Create a two-stage Markov machine presented in Figure 5.9:
**while** k∈{K} **do**
10:    Calculate $n_{e}$ with $n_{o}$, *F* and T.11:    **while** $n\in \{n_{e}\}$ **do**12:        Add Rice propagation model to the upload data.13:        Identify the Markov machine state.14:        Identify whether the upload data is received (power is within the threshold). The flag $flag_{t}$ sets to 0 when the data is lost.15:        Update the Markov machine state.16: 
       **while** the received LHSs have not been fully processed **do**17:              **while** Time of re-sensing is smaller than *R* **do**18:                   Generate AWGN noises with $N_{d}$ length for $N_{n}$ channels.19:                   Calculate the sub-channel number whose power is over the threshold.20:                   **if** More than $N_{k}$ sub-channels detect the busy state. **then**21:                     $T_{sensecount}=T_{sensecount}+1$.22:                     **if** The re-sensing time reaches R **then**23:                          $flag_{s}=0$.24:                     **end if**25:                   **end if**26:              **end while**27:              **if** current opportunity length $L>(T_{sensecount}\;\cdot \;T_{sen}+\delta_{T})$ **then** $flag_{f}=0$.28:              **end if**29:              **if** $flag_{t}$, $flag_{s}$, and $flag_{f}$ equal 1 **then**30:                  Summarize throughput for this opportunity.31:              **end if**32: 
      **end while**33:    **end while**34:
**end while**
35:Calculate the simulation throughput value $Th_{UAV}$.


### 4.2. RA-OLS Communication Demonstration

We consider the case that UAVs fly away from the transmitter with the distance ranging from d=4000 m to d= 40,000 m. The flying speed is constant of 20 m/s. The receiver sensitivity $S_{r}$ at UAV side is −50 dBm. The transmission power received at the reference point $d_{0}$ is 2 watts meaning that $\Omega_{0}=2$. $p_{gg}$ and $p_{bb}$ are configured as 0.995 and 0.96, respectively. The LHSs are generated in terrestrial stations and uploaded to UAVs per every $T_{o}=2$ seconds (distortion of PU patterns caused by the link degradation assumes to be compensated). To avoid the collision towards PUs, we use the required SNR in Table 2 to generate the probability of false alarm for each link. Given the CDR policy applied for the determination of PUs, the detection probability ${\bar{P}}_{D}$ is configured to 0.9. $\lambda_{\Delta T}$ and $\lambda_{b}$ are the parameters to balance the performance and fairness fitting during allocation. For the onboard spectrum sensing, since only the existence of PU patterns is needed, which means we can have a lower sampling frequency and sample number for the detection. We configure $F_{s}=2000$ Hz and $T_{sen}=0.01$ in this case, hence we have $N=F_{s}\;\cdot \;T_{sen}=20$ samples for detecting the PU presence. The maximum re-sensing time *R* in the E-MAC layer (discussed in Section 2.2) is 3. The maximum number of link opportunities $n_{o}$ (discussed in Section 2.1) is 4. The detailed configuration table is presented in Table 3.

#### 4.2.1. Single UAV

**Figure 7:**
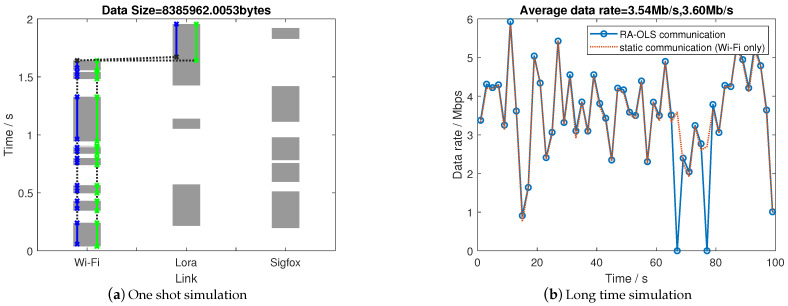
Communication performance with single UAV operation.

For the single UAV operation, a demonstration of using the RA-OLS scheme in terms of spectrum utilization condition is plotted in Figure 7a, where the green lines represent theoretical access behavior, and the blue lines represent practical access. The details for Figure 7a are presented in Table 4 (table contents are unified with the format: start time stamp—end time stamp/s). We repeat the simulation for 50 times where PU patterns are randomly generated, the long-term simulation result is presented in Figure 7b.

As depicted in Figure 7a and Table 4, the transmission through Wi-Fi is preferable because of its high-throughput (Wi-Fi outperforms over 5000 times than Lora and Sigfox in Table 2). Therefore, the sufficient utilization of Wi-Fi is enabled firstly followed by using the remaining links (use Lora in the $10_{th}$ opportunity) to mitigate the time gap. The $6_{th}$ opportunity is filtered by the E-MAC due to the short opportunity length (the minimum duration is 0.02 s). Such latency is also observed in the practical start time (practical access time has an averaged time delay for 0.02 s). Consequently, by adopting such dynamic communication scheme, the proposed RA-OLS scheme is promising to coordinate with multiple links and improve the communication performance by hopping between links.

When observing the long-term data rate results in Figure 7b, the RA-OLS scheme reaches or slightly outperforms the static scheme by using Wi-Fi only (best link) if the transmission dropouts are negligible. The reason of minor superiority to static scheme is the low throughput among Lora and Sigfox. The dropout is introduced by the transmission failure of LHSs ($\pi_{b}=0.1111$ in this case). The uplink degradation effect on the communication is further analyzed in the sensitivity section.

#### 4.2.2. Multiple UAVs

**Figure 8:**
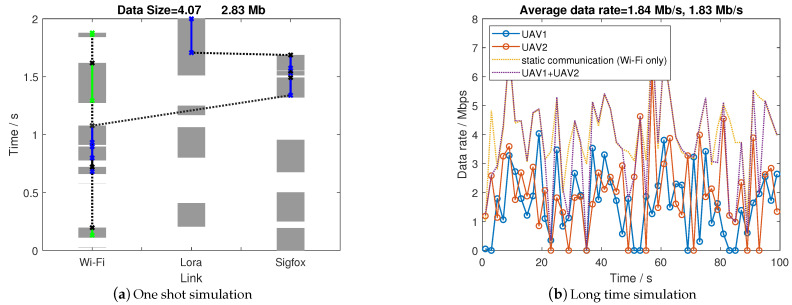
Communication performance with two UAVs operations.

We consider the RA-OLS communication scenario using two UAVs in Figure 8a. It is shown that the Wi-Fi link is sufficiently assigned and shared among two UAVs. To make up with the less usage of Wi-Fi for the second UAV (blue), additional Lora and Sigfox links are used. The reason that the second UAV does not start from the earlier time is the closest opportunity in the Wi-Fi link remains the highest cost function value because of significant high throughput.

**Figure 9:**
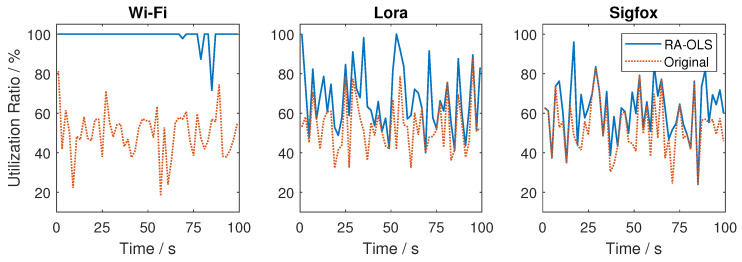
Utilization rate among individual links with K=2 UAVs. (The blue solid lines are the link utilizations with RA-OLS, while the orange virtual lines represent the initial link occupation without transmissions).

When observing the long-term simulation result in Figure 8b, the transmission among two UAVs are balanced in equal with similar data rate, which reveals the effectiveness of using the cost function structure to manage spectrum among two UAVs. However, the Wi-Fi link may not always be assigned entirely leading to an extreme low data rate for the UAV which has not been allocated with Wi-Fi (the cause of extremely small data rate values). By counting up the data rate in two UAVs, the summarized data rate is close or over to the static communication rate with Wi-Fi link only when ignoring the transmission loss (static communication could also have transmission losses caused by the non-ideal detectors). This phenomenon means that the Wi-Fi is largely used in the long-term simulation, which can be further validated by plotting the link utilization figures in Figure 9. It is noting that Wi-Fi has not fully used especially at 85 s, and we attribute it to the sub-optimal allocation solution in the link assignment.

### 4.3. Saturation Throughput Analysis

This section aims at investigating the gap between the proposed theoretical model (19) in Section 3.1.3 and the practical communication simulation in Section 4.1 due to the unknown allocation performance. To mitigate the diversity among links and simplify the analysis, this section simulates multiple homogeneous link types with the Lora configuration (see Table 2). The data rate for each link is normalized to 1 b/s. The iterative duration time is extended to $T_{o}=5$ seconds to enable the simulation of large UAVs *K* operations in the spectrum scarce environment (small link number *M*). We estimate the coefficient weights in (19) with values of $C_{a}=10$ and $\lambda_{Ca}=0.15$. We set the following configurations as the reference, for instance, $\lambda_{\Delta T}=0.8$, $\lambda_{b}=0.1$, $\lambda_{L}=10$, $\lambda_{Q}=10^{1}$ and $n_{o}=2$. The UAVs are randomly deployed with the distance ranging from 7000 to 11,000 m (following a unified distribution) in accordance with the packet dropout rate ranging in [0.16,0.77]. Other configurations remain the same in Table 3.

**Figure 10:**
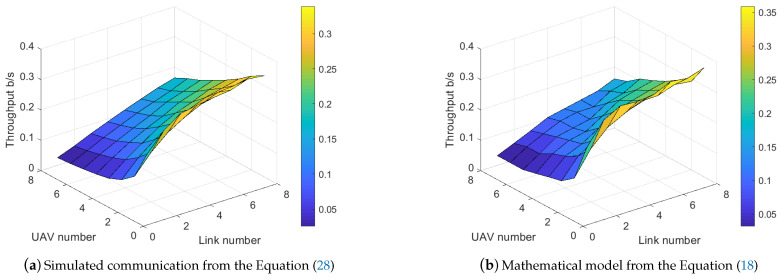
Saturation throughput per UAV derived from the simulated data and the mathematical model.

By repeating the simulation for 500 times and differing the UAV number and link number in [1,8] and [1,8], respectively, the saturation throughput surface planes are presented in Figure 10.

By observing the throughput planes enabled with the RA-OLS in Figure 10, the throughput can be improved by reducing the UAV number *K* and increasing the link number *M*. Furthermore, the degradation of throughput tends to be faster with smaller *K* values and *M* values, whilst the drop rate tends to be constant with big *K* value. We attribute such phenomenon to the non-linearity in the spectrum dense environment. Moreover, the presented mathematical model approximates the simulated communication in terms of the non-linearity and values. The uneven surface plan in Figure 10b is caused by the discretization of $n_{e}$ when estimating the packet dropout rate.

**Figure 11:**
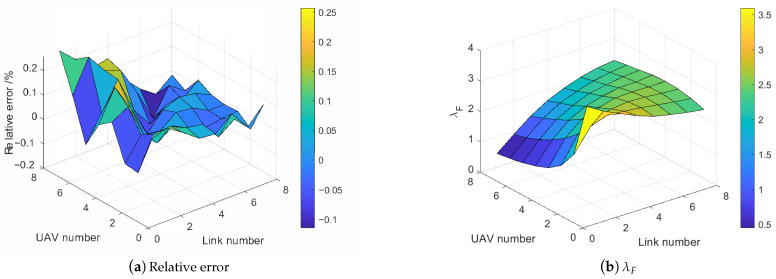
Communication performance surface from the simulated data and the mathematical model.

For the further analysis of the saturation throughput error between Figure 10a,b, We define the relative error function: $e=\frac{{\hat{Th}}_{UAV}-{Th}_{UAV}}{{Th}_{UAV}}$, where ${Th}_{UAV}$ is the simulated throughput value and ${\hat{Th}}_{UAV}$ is the mathematical throughput. The relative error plane figure is presented in Figure 11a. As is shown, the relative error $e_{th}$ is limited within 20% for most cases (the mean of $e_{th}$ is 13.81%). The error turns to be more constant in the spectrum dense (M>K) environment, whilst the error variance grows when M<K. Similar with the uneven reason in Figure 10b, we attribute such error to the mutation when discretizing $n_{e}$ especially when $n_{e}$ is small and $n_{o}$ is big.

It is noting that the coefficient weights aiming at adjusting the degradation and average value of the surface for M>K are effective by having small relative errors (less than 0.1). We remain the same configuration for the remaining simulations.

Given the fact that the RA-OLS mathematical model relies on a pre-knowledge of the allocated opportunity number factor $\lambda_{F}$, we plot the $\lambda_{F}$ plane versus UAV number *K* and the link number *M* in Figure 11b to reflect the hidden coherence among $\lambda_{F}$, *K*, and *M*. It is depicted in Figure 11b that $\lambda_{F}$ shows the biggest value when M=K, and declines either in the spectrum dense environment M>K or the spectrum scarcity M<K. The cause of dropping $\lambda_{F}$ when M<K is that UAVs tend to possess fewer resources when lacking resources. The cause of dropping $\lambda_{F}$ when M>K is that better opportunities (longer duration in this case) are used leading to the unacceptance of poor opportunities so that to save the opportunity number. Moreover, the spectrum scarcity issue shows more impact on the $\lambda_{F}$ providing the faster decline rate with smaller *M* and larger *K*.

#### 4.3.1. Sensitivity to Packet Drop Via Uplinks

Given the parameters relevant to the packet drop rate $P_{d}$, i.e., distance between UAVs and GCS *d*, reference distance $d_{0}$, reference transmission power $\Omega_{0}$, receiver sensitivity $s_{r}$, and good state and bad state probabilities $p_{gg}$ and $p_{bb}$, this section performs the sensitivity analysis in terms of packet drop rate $P_{d}$, relative error *e* and throughput by varying some of configurations. For instance, *d* ranges from 6000 m to 18,000 m with a constant interval of 4000 m; receiver sensitivity ranges from 6 × 10${}^{-9}$ to 1.8 × 10${}^{-8}$ with a constant interval of 4 × 10${}^{-9}$; and $p_{gg}$ ranges from 0.985 to 0.995 with an interval of 0.005. The UAV number *K* and link number *M* are set to 5, respectively (the packet drop rate shows fixed impacts with various *K* and *M*). We calculate the relative error for $N_{d}$ which is the opportunity number received successfully by UAVs during $T_{o}$, and UAV throughput by repeating simulation for 1000 times (the relative errors are denoted as $e_{nd}$ and $e_{th}$). Other parameters remain the same discussed in Section 4.3. The thorough sensitivity table caused by the uplink packet drop factor is presented in Table 5.

As shown in Table 5, both $e_{nd}$ and $e_{th}$ tend to present a small value (around 10%) with various $P_{d}$ values, which means the developed finite state machine and the mathematical model is robust against failures in upload links.

By observing the distance *d* effect on $P_{d}$ and $Th_{UAV}$, with the current configuration ($d_{0}=1$, $\Omega_{0}=2$), $P_{d}$ starts increasing from $1-\pi_{g}=0.1111$ at 4000 m and reaches to 1 after 14,000 m. The growth rate of $P_{d}$ increases the most rapidly during 6000 to 10,000 m ($P_{d}$ doubles when *d* increases by 2000 m). In the meantime, with the growing of $P_{d}$, the practical UAV throughput declines from 0.31 at 4000 m to 0.0071 at 14,000 m. The decline rate tends to increase till the throughput shows a small value (the maximum decline rate is around 6 times at 14,000 m).

When observing the receiver sensitivity $s_{r}$ effect on $P_{d}$ and $Th_{UAV}$, the $P_{d}$ shows similar growing tendency with the increment of $s_{r}$ as the *d* effect. The maximum growth rate of $P_{d}$ is also around 2 times when $s_{r}=7\times 10^{-9}$. Similarly, the decline of UAV throughput speeds up when $s_{r}=4\times 10^{-9}$ and slows down when $s_{r}=1.3\times 10^{-8}$.

By reducing the $p_{gg}$ value, i.e., the possibility of maintaining good states in the uplink receiver, the $P_{d}$ increase along with the degradation of $Th_{UAV}$. Especially, the improvement of $Th_{UAV}$ and decline of $P_{d}$ are dominant when $p_{gg}$ increase from 0.795 to 0.995 by 20%. $Th_{UAV}$ and $P_{d}$ remain relatively stable when $p_{gg}<0.795$.

#### 4.3.2. Sensitivity to Spectrum Sensing

The spectrum sensing configurations are: theoretical detection probability $P_{D}$, received signal strength from unknown PUs $\delta_{s}$, noise strength $\delta_{n}$, sampling rate $F_{s}$, parameters in sensor fusion policy $N_{n}$ and $N_{k}$. Some other configurations relevant to the time delay caused by spectrum sensing, e.g., *R* and $T_{sen}$ are evaluated in the following section. It is noting that the $\delta_{s}$ denotes the PU strength which is randomly updated every $T_{o}$ seconds, hence the propagation model in PUs is not considered. For analyzing the spectrum sensing impacts on the UAV throughput, we select parameters of $P_{D}$, $\delta_{s}$ and $N_{k}$ for the evaluation. We define $F_{pf}=P_{F}^{R}$, and $e_{th}$ for representing the relevant errors for $Th_{UAV}$. The sensitivity table is presented in Table 6.

As depicted in Table 6, the more strict $P_{D}$ value causes bigger probability of false alarm ${\hat{P}}_{F}$ leading to smaller UAV throughput. The increment rate of ${\hat{P}}_{F}$ slows down with the growing $P_{D}$, whilst the degradation of $Th_{UAV}$ speeds up especially when $P_{D}>0.96$. Both the theoretical ${\hat{F}}_{pf}$ and practical $F_{pf}$ increase. Theoretical ${\hat{F}}_{pf}$ shows a bigger growing rate when $P_{D}>0.945$ than $F_{pf}$, and maintains a higher steady possibility value of 0.91. However, the presented model may fail under big $P_{F}$ environment because of having the large $e_{th}$ result when $P_{D}=0.945$, although the UAV throughput is extremely small.

Similar results are obtained when observing the SNR impact. Nevertheless, $N_{k}$ tends to have stronger negative influence. For instance, the relative error $e_{th}$ still remains 0.88 when $N_{k}=3$ with ${\hat{P}}_{F}=0.6359$, whilst the $e_{th}$ is small (−0.1611) when PD=0.945 with the similar ${\hat{P}}_{F}=0.5385$. Therefore, the hard fusion model could bring more errors (may be resulted from the quasi random generation method when simulating AWGN noises) especially when $N_{k}$ is relatively small.

#### 4.3.3. Sensitivity to E-MAVLink and E-MAC

Given the two primary configurations in E-MAVLink and E-MAC, i.e., the maximum re-sensing number *R* and the maximum opportunity number per LHS $n_{o}$, we plot the following sensitivity table by differing *R* and $n_{o}$ values. $P_{D}$ is increased to 0.93 to amplify side effects in ${\hat{P}}_{F}$. Other default configurations are presented in Section 4.3.

As shown in Table 7, the rising of *R* decreases the probability of false alarm effect and improves the UAV throughput by around 60%. Especially the performance is significantly improved when *R* is increased from 1 to 3. The errors between theoretical model and practical simulations for $Th_{UAV}$ and $F_{pf}$ are constant, which also validates the effectiveness in modeling R effect.

When observing the $n_{o}$ impact on the communication, there is no clear tendency in terms of UAV throughput $Th_{UAV}$, in this simulation. There is a slightly decline in ${\hat{e}}_{nd}$ resulted from discretization. However, the less segment number with longer length could result in high interference.

### 4.4. RA-OLS Performance with IF

**Figure 12:**
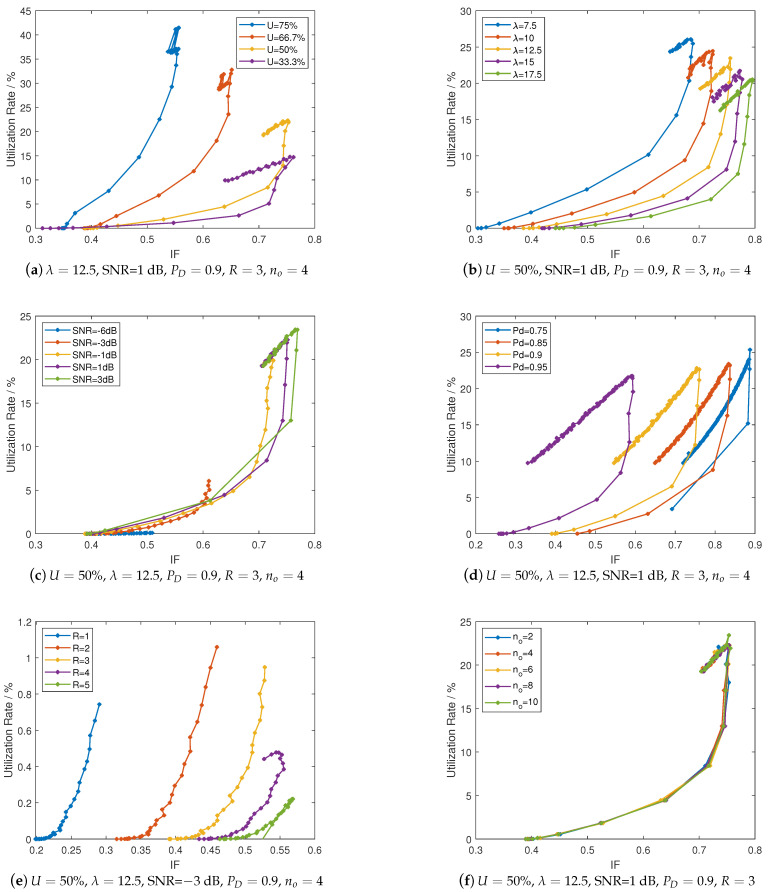
Utilization rate versus interference factor by differing $T_{sen}$ with M=6 links and K=3 UAVs.

Regarding several parameters exist in the model bringing difficulty in choosing the favorable values, as well as the selection of values essentially is a trade-off problem, this section simulate the RA-OLS communication versus IF factor (23) (the LHS length is reflected in IF). We use the definition of $U_{i}$ in Section 4.1 to measure the density of link opportunities and $\lambda_{v}=\lambda_{a}=\lambda$ to denote the length of link opportunities in the PU pattern factor. We use SNR and ${\bar{P}}_{D}$ as the parameters to characterize the spectrum sensing failure. Time effect in the E-MAC is measured by differing *R* number, along with varying $T_{sen}$ in the range of [0.001,0.03]. We use $n_{o}$ to differ the E-MAVLink length. The configurations, e.g., $U_{i}=50\%$, λ=12.5, SNR=1 dB, $P_{D}=0.9$, R=3 and $n_{o}=4$ are selected as the benchmark value along with other configurations presented in Section 4.1. The results of the utilization rate *U* obtained from Equation (27) versus IF are presented in Figure 12.

As depicted in Figure 12, all the *U* versus IF curves show an initial upward trend, i.e., having an increasing growth with the increment of $T_{sen}$ till the curves reach to a peak. The cause of having such peaks is that the over-large $T_{sen}$ value may waste more time in sensing leading to low-efficiency in transmission.

By observing the peak values of *U* versus IF curves in Figure 12, some results can be obtained. $U_{i}$, λ, SNR, and *R* show a significant impact on the *U* vertical ordinates. The peak in *U* for the $P_{D}$ figure changes slightly, which means that the side effects of the $P_{D}$ can be compensated by configuring a bigger $T_{sen}$ value.

In specific, as illustrated in Figure 12a,b, longer (bigger $U_{i}$) and more dense (bigger λ) link opportunities enable a more efficient communication performance (higher *U* and smaller IF). When analyzing the impact from the spectrum detector on the performance (see the Figure 12c,d, the stronger signal power received by UAVs could reduce the $T_{sen}$ value corresponding to the peaks. The increasing $P_{D}$ also improves the communication by reducing the IF at a cost of bigger $T_{sen}$ for maintaining the $P_{D}$. In Figure 12e, the re-sensing scheme, i.e., the *R* effect improves the communication performance with small $T_{sen}$, whilst the peak value degrades when *R* increases. As shown in Figure 12f, the $n_{o}$ presents limited small impacts on both utilization rate and IF according to the derived models.

In accordance with above results in Figure 12, the favorable selection of configurations can be obtained following the procedure. When we have the PU pattern configurations, i.e., $U_{i}=50\%$ and λ=7.5, a preferred $T_{sen}$ can be obtained by selecting the peak values from curves (around 0.0013). A preferred SNR = 1 dB (relevant to the threshold selection) is obtained from Figure 12c, as well as having $P_{d}=0.095$ because of possessing higher utilization rate and small IF values. Moreover, *R* can be chosen to 5 owing to the largest peak value when $T_{sen}=0.0013$.

### 4.5. Performance Comparison

In this section, we use the static communication scheme (see the Section 3.3) without having the dynamic access mechanism as the benchmark for the performance comparison purpose, as well as using the definition of utilization rate *U* in the Equation (27) for measuring the performance. We use Wi-Fi configurations presented in Table 2. The implementation of the static link is similar to the RA-OLS discussed in Section 4.1, whilst only the determined links are allocated to its corresponding UAVs to prevent having the link-hopping mechanism.

**Figure 13:**
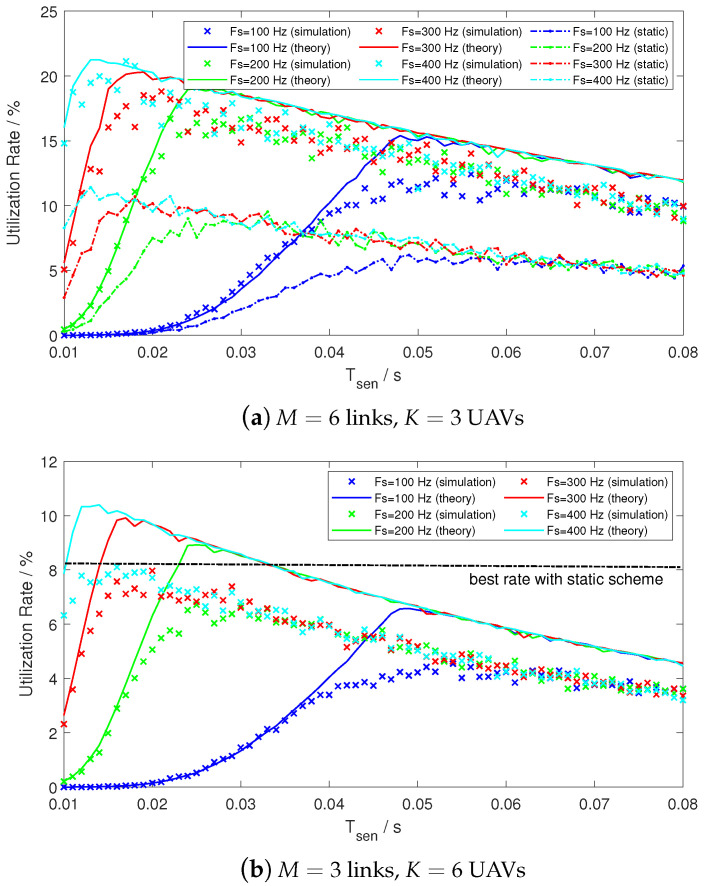
Utilization rate versus sensing time and $F_{s}$ when SNR=1.

The probability of detection is reconfigured as ${\bar{P}}_{D}=0.9$ to amplify the side effect resulted from non-ideal detectors. Considering the link degradation effect, the SNR at the onboard spectrum detector side is set to 1 dB. We simulate the communication with static scheme discussed in Section 3.3 and calculate the throughput in statistic. By differing sensing time $T_{sen}$ and sampling rate $F_{s}$ in the airborne detectors (see the Section 3.1.2), the comparison figure of the spectrum utilization rate *U* versus $T_{sen}$ is illustrated in the Figure 13, where *U* is averaged by *K* UAVs.

As depicted in Figure 13a, it is observable that the performance with the RA-OLS scheme outperforms the static link scheme for nearly 50% under the spectrum dense environment when M>K. Providing that the static scheme is invalid when M<K, we apply the best utilization assumption of U=50%/6=8.3% from the Equation (26) as the benchmark. By observing Figure 13b, the RA-OLS enabled communication still outperforms the static link scheme with small $T_{sen}$ value under the spectrum scarce environment.

Moreover, with the increment of $T_{sen}$, the *U* curve increases rapidly and declines gradually. The reason for having the upward tendency is that the probability of false alarm ${\hat{P}}_{F}$ becomes smaller and the link opportunities are more likely to be detected in the air. With the further growth of $T_{sen}$, *U* declines on account of consuming more time in the E-MAC processing. By increasing the $F_{s}$ values, the peak of *U* moves towards a smaller $T_{sen}$. The sample number $N=F_{s}\;\cdot \;T_{sen}$ is determined around 5 samples in this case, but the optimal *N* value also relates to other parameters, such as SNR, $N_{k}$, $\delta_{T}$, and $\lambda_{F}$ discussed in Section 4.4.

It worth noting that the capacity of RA-OLS for Figure 13b is 67 according to (25), whilst the capacity for the static scheme is only 3 because of the assumption of one user per link.

**Figure 14:**
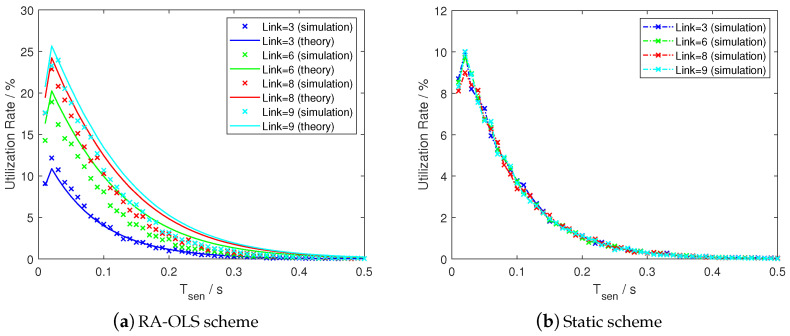
Spectrum utilization rate by differing link number with K=3 UAVs, SNR=1 dB, and $F_{s}=400$ Hz.

Furthermore, we compare the performance between the RA-OLS and the static scheme by differing the link number *M* and the result is presented in Figure 14. A similar result that the proposed mathematical throughput model is valid for measuring the simulated communication scheme can be obtained by having small absolute errors, i.e., less than 3% in Figure 13a and 2% in the Figure 13b.

As obtained in Figure 14, the RA-OLS and static schemes show the similar *U* results when M=K=3. The *M* value does not affect the *U* values with the static scheme (see Figure 14b, whilst the *U* with RA-OLS scheme improves with the increment of *M* (see Figure 14a, which reflects the advantages of using RA-OLS scheme on improving the communication performance. By observing the peak values in Figure 14a, the peak of *U* tends to slow down with the growing *M*, which also fits the tendency observed in Section 4.3.

## 5. Conclusions

For providing dynamic communication schemes among multiple UAVs and addressing the spectrum scarcity problem, this paper proposes an RA-OLS enabled UAV communication solution which is capable of supporting swarm operations under the spectrum scarce environments. The spectrum resources are coordinated by ground stations, and uploaded to UAVs. Technical solutions, such as the E-MAVLink and E-MAC layer design are considered, along with the mathematical saturation throughput model denoted for RA-OLS. The IF factor is applied for analyzing the interference extent to other PUs. By demonstrating some typical links, e.g., Wi-Fi, Lora, and Sigfox for single UAV and multiple UAVs, the proposed RA-OLS shows an improvement in utilizing link resources and throughput. The fine-tuned piece-wise throughput model proves to enable the approximation of the simulated communication behaviors with relative errors around 10%. The effects of PU traffic patterns, non-ideal detector, the re-sensing scheme in the E-MAC, and E-MAVLink length on the RA-OLS performance are analyzed with some results highlighted. For instance, the longer and more dense link opportunity in the PU traffic pattern enables an efficient communication; the side effect from the $P_{D}$ resulted by the time delay in non-ideal detectors can be compensated by having a bigger $T_{sen}$; the re-sensing scheme alleviates the $T_{sen}$ value at the cost of degradation of the peak value of *U*; and $n_{o}$ presents limited impact in terms of utilization rate and IF. Additionally, by comparing with the static communication scheme, the utilization rate for RA-OLS is improved by over 50% than the static scheme with sufficient link resources, and the utilization rate is greater than the best rate with the static scheme by nearly 20%.

## Figures and Tables

**Table 1:** Explanation of E-MAVLink frame.

Acronym	Description
STX	State-of-test marker for indicating start of package.
LEN	Length of payload.
INC	Incompatibility flag for compatibility of receivers.
CMP	Compatiblity flag.
SEQ	Number of packet sequence.
SYS	Identification of system.
COMP	Identification of components sending messages.
MSG	Identification of message type in payload.
PAYLOAD	Messages.
CKA/B	Checksum.
SIG	Signature of package for security purpose.
NLHS	Number of the segmented LHS. (0 × 00 $-\;n_{o}$)
SPAYLOAD	Message of LHS.

**Table 2:** Link parameter configurations.

Frequency	Network Type	Technology	$N_{n}$	$N_{k}$	Throughput bps	$\lambda_{a}$	$\lambda_{v}$	Minimum SNR
2.4–2.49 GHz MHz	WLAN	Wi-Fi	13	4	10 M	9	9	10 dB
868–870 MHz	LPWAN	Lora	17	6	2 k	6	6	−2 dB
902–928 MHz	LPWAN	SigFox	8	3	11 k	7	7	−1 dB

**Table 3:** PU traffic parameters and other parameters configurations.

Parameter	Value	Parameter	Value
$T_{o}$	2 s	$\lambda_{\Delta T}$	0.6
$\lambda_{b}$	0.1	ϵ	0.9
$\lambda_{L}$	10	$\lambda_{Q}$	$10^{-7}$
$F_{s}$	2000	$T_{sen}$	0.01 s
$\delta_{T}$	0.01 s	${\bar{P}}_{D}$	0.9
*R*	3	$n_{o}$	4

**Table 4:** Probability of false alarm with its threshold value over links.

	1	2	3	4	5	6	7	8	9	10
Wi-Fi	0.04–0.24	0.35–0.43	0.50–0.56	0.74–0.80	0.84–0.89	0.90–0.90	0.94–1.33	1.48–1.54	1.56–1.64	N/A
Lora	0.22–0.57	1.05–1.14	1.43–1.96	N/A	N/A	N/A	N/A	N/A	N/A	N/A
Sigfox	0.20–0.51	0.59–0.76	0.78–0.98	1.11–1.42	1.83–1.92	N/A	N/A	N/A	N/A	N/A
Theory	Wi-Fi 0.04–0.24	Wi-Fi 0.35–0.43	Wi-Fi 0.50–0.56	Wi-Fi 0.74–0.80	Wi-Fi 0.84–0.89	Wi-Fi 0.90–0.90	Wi-Fi 0.94–1.33	Wi-Fi 1.48–1.54	Wi-Fi 1.56–1.64	Lora 1.64–1.96
Access	Wi-Fi 0.06–0.24	Wi-Fi 0.37–0.43	Wi-Fi 0.52–0.56	Wi-Fi 0.76–0.80	Wi-Fi 0.86–0.89	N/A	Wi-Fi 0.96–1.33	Wi-Fi 1.5–1.54	Wi-Fi 1.58–1.64	Lora 1.67–1.96

**Table 5:** Sensitivity table of performance to packet dropout rate parameters.

d/m	4000	6000	8000	10,000	12,000	14,000
${\hat{P}}_{d}$	0.1136	0.1431	0.2886	0.5939	0.8707	0.9799
$Th_{UAV}$	0.3083	0.3044	0.2506	0.1457	0.0449	0.0071
$e_{nd}$	0.1064	0.0804	0.1004	0.0674	0.0827	0.0839
$e_{th}$	0.1450	0.1210	0.1304	0.1099	0.1460	0.1232
$s_{r}$	1 × 10${}^{-9}$	4 × 10${}^{-9}$	7 × 10${}^{-9}$	10${}^{-8}$	1.3 × 10${}^{-8}$	1.6 × 10${}^{-8}$
${\hat{P}}_{d}$	0.1118	0.1556	0.3351	0.5939	0.8046	0.9232
$Th_{UAV}$	0.3165	0.3055	0.2397	0.1454	0.0702	0.0268
$e_{nd}$	0.0865	0.0712	0.0691	0.0850	0.0702	0.1317
$e_{th}$	0.1176	0.1006	0.1049	0.1126	0.0959	0.1417
$p_{gg}$	0.495	0.595	0.695	0.795	0.895	0.995
${\hat{P}}_{d}$	0.9665	0.9589	0.9470	0.9254	0.8740	0.5939
$Th_{UAV}$	0.0120	0.0146	0.0203	0.0291	0.0438	0.1438
$e_{nd}$	0.0811	0.1088	0.0437	0.0085	0.0991	0.0844
$e_{th}$	0.1087	0.1215	0.0405	0.0214	0.1466	0.1245

**Table 6:** Sensitivity table of performance to spectrum sensing parameters.

$P_{D}$	0.9	0.915	0.93	0.945	0.96	0.975
${\hat{P}}_{F}$	0.0787	0.1618	0.3108	0.5385	0.8002	0.9700
$Th_{UAV}$	0.1413	0.1376	0.1341	0.1144	0.0815	0.0300
${\hat{F}}_{pf}$	0.00049	0.0042	0.0300	0.1562	0.5123	0.9126
$F_{pf}$	0.0092	0.0277	0.0734	0.1852	0.4295	0.7808
$e_{th}$	0.1447	0.1707	0.1703	0.1611	−0.0922	−0.5682
SNR	−5	−4	−3	−2	−1	0
${\hat{P}}_{F}$	0.9862	0.8954	0.5259	0.0787	0.00064	$3.5\times 10^{-8}$
$Th_{UAV}$	0.0194	0.0589	0.1147	0.1427	0.1389	0.1440
${\hat{F}}_{pf}$	0.9593	0.7179	0.1455	0.00049	$2.6\times 10^{-12}$	$4.5\times 10^{-25}$
$F_{pf}$	0.8572	0.5772	0.1799	0.0090	$5.2\times 10^{-07}$	0
$e_{th}$	−0.6901	−0.2822	0.1742	0.1331	0.1650	0.1238
$N_{k}$	2	3	4	5	6	7
${\hat{P}}_{F}$	0.8513	0.6359	0.3904	0.1946	0.0787	0.0258
$Th_{UAV}$	0.0223	0.0614	0.1061	0.1316	0.1384	0.1417
${\hat{F}}_{pf}$	0.6169	0.2571	0.0595	0.0074	0.00049	0.0000017
$F_{pf}$	0.8390	0.5490	0.2369	0.0620	0.0096	0.00091
$e_{th}$	1.5861	0.8803	0.4231	0.2202	0.1690	0.1421

**Table 7:** Sensitivity table of performance to E-MAVLink and E-MAC parameters.

R	1	2	3	4	5
$Th_{UAV}$	0.0834	0.1161	0.1321	0.1333	0.1391
${\hat{Th}}_{UAV}$	0.1115	0.1462	0.1570	0.1586	0.1574
${\hat{F}}_{pf}$	0.3108	0.0966	0.0300	0.0093	0.0029
$F_{pf}$	0.4153	0.1731	0.0733	0.0301	0.0131
$n_{o}$	2	4	6	8	10
$Th_{UAV}$	0.1295	0.1385	0.1298	0.1311	0.1363
${\hat{Th}}_{UAV}$	0.1570	0.1570	0.1570	0.1570	0.1570
$e_{nd}$	4.8737	4.8737	4.8737	4.8737	4.8737
${\hat{e}}_{nd}$	4.4724	4.4229	3.8967	3.6275	3.4366

## Data Availability

Not applicable.
